# Foscan® uptake and tissue distribution in relation to photodynamic efficacy

**DOI:** 10.1038/sj.bjc.6600682

**Published:** 2003-01-28

**Authors:** P Cramers, M Ruevekamp, H Oppelaar, O Dalesio, P Baas, F A Stewart

**Affiliations:** 1 Experimental Therapy (H6), The Netherlands Cancer Institute/Antoni van Leeuwenhoek Ziekenhuis, Plesmanlaan 121, 1066 CX Amsterdam, The Netherlands; 2 Biometrics and Statistics, The Netherlands Cancer Institute/Antoni van Leeuwenhoek Ziekenhuis, Amsterdam, The Netherlands; 3 Medical Oncology, The Netherlands Cancer Institute/Antoni van Leeuwenhoek Ziekenhuis, Amsterdam, The Netherlands

**Keywords:** photodynamic therapy, Foscan, pharmacokinetics, vascular damage

## Abstract

Clinical photodynamic therapy (PDT) schedules are based on the assumption that optimum drug–light intervals are times at which there is a maximum differential between photosensitiser retention in the tumour and surrounding normal tissue. However, vascular-mediated effects contribute to tumour destruction by PDT; therefore, plasma sensitiser levels and endothelial cell drug exposure could also be important determinants of PDT response. The purpose of this study was to investigate the influence of tumour, tissue and plasma concentrations of the photosensitiser Foscan® (*meta*-tetrahydroxyphenylchlorin, mTHPC) on PDT response. Groups of BalbC nude mice, bearing human mesothelioma xenografts (H-MESO1) were injected (i.v.) with a single dose of ^14^C-labelled mTHPC, or with two doses, separated by 72 h. Drug levels in plasma, tumour and normal tissues were measured at 5 min to 120 h after drug administration. The PDT tumour and skin responses were evaluated by illuminating separate groups mice at intervals of 5 min to 120 h after injection of Foscan (nonlabelled). Drug levels in both tumour and skin increased during the first 24 h after a single injection, and remained almost constant for at least 120 h. The second injection produced a further, rapid increase in mTHPC levels in tumours and skin, with steady state being maintained from 20 min to 120 h. By contrast, PDT response of both tumours and skin were maximal for illumination at 1–3 h after drug, with very little response when illumination was given 48–120 h after drug. There was no significant correlation between tumour or skin drug level and PDT response. There was, however, a significant correlation between plasma drug levels and tumour or skin response, excluding an initial distribution time of 20 min. These studies demonstrate a pronounced disassociation between tumour drug levels and optimum drug–light intervals for PDT response with Foscan. We suggest that the PDT effect, in both tumours and normal tissues, is largely mediated via vascular damage and that the selectivity of PDT is not based on differential tumour drug uptake.

Photodynamic therapy (PDT) is increasingly used as a treatment for small localised tumours or as an adjuvant to debulking surgery for more advanced disease. The principle of PDT involves administration of a photosensitiser, followed by a distribution interval, and subsequent illumination of the tumour area with light of an appropriate wavelength to excite the sensitiser to its triplet state. The ensuing photochemical reaction is dependent on the presence of oxygen and generates singlet oxygen and free radicals. These toxic species are highly reactive and short lived; therefore, the resultant tissue damage occurs very close to the site of production.

The photosensitiser used in this study, *meta*-tetrahydroxy-phenylchlorin (mTHPC, trade name Foscan®), is a pure chemical with a strong absorption peak in the red part of the spectrum (652 nm). Foscan is one of the most potent photosensitisers currently available for clinical use. Clinical protocols for PDT are based on the assumption that optimum intervals between photosensitiser administration and illumination are times at which there is a maximum differential between drug retention in the tumour and in surrounding normal tissue. For Foscan-PDT, a drug–light interval of 4 days is usually chosen. The relation between optimum drug dose, light dose and drug–light interval is, however, complex and there is little systematic data available to relate these parameters to tumour response and normal tissue toxicity.

Distribution studies in experimental animals have demonstrated that Foscan levels increase in tumours from 1 to 3 days after injection, whereas drug levels in many normal tissues remain constant or decrease over the same period (Alian *et al*, 1994; Peng *et al*, 1995; Whelpton *et al*, 1995,^1996^; Veenhuizen *et al*, 1997a). This leads to differential drug levels in tumour *vs* normal tissue at 2–4 days after injection. Early clinical data in mesothelioma patients also demonstrated good selectivity, with drug concentrations in the tumour of 5–14 times that in surrounding normal tissue (pulmonary artery, bronchus, muscle and skin) at 2–3 days after drug administration (Ris *et al*, 1991; Braichotte *et al*, 1992). A study of PDT for metastatic gynaecological cancer (Wierrani *et al*, 1997) also demonstrated concentration ratios of 3–6 for tumour/fatty tissue (retroperitoneal pelvic wall area) in a series of patients biopsied at 4 days after Foscan. However, despite the generally impressive differentials seen between tumour and normal tissue sensitiser levels, a truly selective tumour necrosis is seldom seen after PDT.

Tumour destruction by PDT can occur either as a direct result of tumour cell killing or as a secondary consequence of vascular collapse (Henderson *et al*, 1985; Star *et al*, 1986). The endothelial cell has been identified as an early target for PDT damage and there is abundant evidence for the importance of vascular-mediated effects after *in vivo* PDT using Foscan and other photosensitisers, like Photofrin (Fingar *et al*, 1990,^1992^). In the light of this information, it would seem logical to shift the emphasis in clinical PDT from targeting the tumour cell to targeting the vasculature. The consequences of vascular damage are predicted to be much more severe for tumours than for normal tissues, which have a more robust and extensive vascular network.

Several experimental studies have identified a discrepancy between times of maximal tumour drug uptake and optimum illumination intervals for the best tumour effect, both for Foscan and for other photosensitisers (Ris *et al*, 1993; Morlet *et al*, 1995; Veenhuizen *et al*, 1997a,1997b) . Optimum illumination intervals are usually considerably shorter than times for maximal loading of the tumour with sensitiser. We hypothesise that it is exposure of the endothelial cells of vessels feeding the tumour to the sensitiser that determines tumour response, and that this is more closely reflected by plasma levels than by tissue levels of photosensitiser. If a correlation between plasma drug levels and tumour response is a general phenomenon, then drug–light intervals of a few hours could be considered for some clinical protocols.

Short drug–light intervals will probably not be suitable for large surface area PDT, where normal tissue toxicity is dose-limiting. However, many applications of PDT involve focal illumination of relatively small areas, and it has even been argued that necrosis of a margin of normal tissue surrounding the tumour is required to achieve cure. Illumination at shorter, more effective, time intervals might allow the drug dose to be lowered, thus reducing the risk of generalised skin photosensitivity. To achieve an optimal PDT effect, the sensitiser dose, time interval between drug injection and illumination, and the light dose should be finely titrated. A greater understanding of the relation between plasma/tissue drug levels and tumour response or normal tissue toxicity would facilitate such a process.

In this study, Foscan distribution at different times after drug delivery is compared with the PDT response of the tumour and normal skin for illumination at the same time points.

## MATERIALS AND METHODS

### Animals and tumour model

All experiments were carried out in accordance with protocols approved by the local experimental animal welfare committee and conformed to national and European regulations for animal experimentation. Female nude BalbC mice (weighing 21–30 g, at an age of 12–26 weeks) were used for all experiments. H-MESO1 (human mesothelioma xenograft) tumour fragments from a subcutaneous donor tumour were implanted on the lower dorsum. Tumour growth was documented twice a week using callipers. The untreated tumours had a volume doubling time of about 15 days. Pharmacokinetic measurements or PDT were performed when tumours had reached a maximum diameter of 8 mm and a depth of at least 3 mm.

### Determination of ^14^C-mTHPC levels in tissues

Mice (six per group) were killed 5 min to 120 h after an intravenous injection (tail vein) of ^14^C-labelled mTHPC (0.3 mg kg^−1^), dissolved in ethanol, polyethylene glycol 400 (PEG) and water (2 : 3 : 5 volume ratio). The ^14^C-mTHPC was provided by Scotia Pharmaceuticals Ltd (Stirling, Scotland) and synthesised by American Radiolabelled Chemicals Inc. (St Louis, USA). Plasma was obtained by exsanguination of the mice and subsequent centrifugation. The following organs were then excised: tumour, skin, lung, heart, liver, kidney, fat (i.p.), muscle, tongue, oesophagus and diaphragm. After rinsing with saline and blotting dry, the organs were cut into 2–3 samples, each weighing <100 mg, and placed in preweighed counting vials. Additional experiments were done in a separate group of mice, perfused via the left ventricle with 5 ml of saline (0.9% NaCl) to remove circulating blood from the organs prior to their removal and counting. A measure of 1 ml of Soluene®-350 (Packard Instrument Company, Groningen, The Netherlands) was added to the samples and vials were stored for 2–3 days until the tissue was dissolved. Ultima Gold MV counting fluid (15 ml) was then added to each vial before counting in a Packard tricarb liquid scintillation counter.

In a second experiment, two injections of ^14^C-labelled mTHPC (0.3 mg kg^−1^) were given to each mouse, separated by 72 h. This was done in order to produce a plasma peak at the time of maximum tumour loading. The animals were again killed at 5 min to 120 h after the second injection, and plasma and tissue samples were collected as described above. ^14^C-mTHPC levels in the plasma and tissues were determined as a percentage of the injected dose per gram tissue or per millilitre plasma. Mean values (±1 s.d.) for groups of six mice were calculated. A second-order exponential decay (*y*=*y*0+*A*1 exp(−*x*/*t*1)+*A*2 exp(−*x*/*t*2)) was fitted to the experimental data to obtain time constants (*T*_1/2_) for the clearance of mTHPC from plasma and various organs.

### PDT treatment of tumours

Tumour-bearing mice (eight per group) were injected intravenously with 0.3 mg kg^−1^ Foscan® (kindly provided by Scotia Pharmaceuticals, Stirling, Scotland). The drug was dissolved in ethanol, PEG and water, as described above. At 5 min to 120 h after injection, mice were illuminated with red light of 652 nm wavelength, while held in restraining jigs. A diode laser (Applied Optronics Corp., South Plainfield, NJ, USA) was used to deliver a light bundle of 12 mm diameter to the tumour area, via quartz fibres with lens applicators (Medlight S.A. Ecublens, Switzerland). The rest of the mouse was shielded. A fluence rate of 100 mW cm^−2^ was used to deliver light doses of 5, 10 or 30 J cm^−2^ to the tumour surface. In a second experiment, two injections of Foscan (2×0.3 mg kg^−1^) were given, separated by 72 h, and tumours were illuminated with 30 J cm^−2^ at 5 min to 72 h after the second injection.

Tumour size was measured twice per week and the time to recurrence was calculated as the time taken for a tumour to increase by >2 mm from a treatment size of 6±0.5 mm geometric mean diameter. This represents approximately a 2.5-fold increase in tumour volume. Tumours that did not regrow within 120 days were defined as cures and assigned a maximum tumour-free survival of 120 days for graphical presentation of the results. Cured mice, and a small number of animals (<5%), which had to be killed because of sickness before 120 days, but without recurrent tumours, were entered as censored observations in the statistical comparison of groups.

### PDT treatment of the normal skin

Animals (six per group) were held in restraining jigs and a skin patch of 13×8 mm^2^ on the lower dorsum was illuminated with 652 nm red light. The rest of the mouse was shielded. Superficial illumination was given (with the diode laser described above) 5 min to 120 h after i.v. tail injection of Foscan (0.3 mg kg^−1^). Light doses of 5 or 10 J cm^−2^ were delivered at a fluence rate of 100 mW cm^−2^. Control animals received no mTHPC prior to illumination, or illumination with light of an unsuitable wavelength (675 nm) at 24 h after Foscan.

Skin damage was evaluated three times per week, by two independent observers, according to a semiquantitative scale (Table 1

**Table 1:** Scoring system for skin reactions

Grade 0:	No change in skin colour
Grade 1:	Minimal redness
Grade 2:	Redness
Grade 3:	Deep blue coloration, wound
Grade 4:	Scab formation

). The percentages of grade 0–4 reactions for each skin patch were calculated to give the mean skin score over the whole treated area. The average skin score over a follow-up time of 30 days was then calculated.

### Statistics

Comparisons of tumour responses between groups were tested using the Breslow rank test, which is a generalisation of the Wilcoxon rank test that allows inclusion of censored observations and the comparison of more than two groups. A comparison of average skin reactions according to drug–light interval was done using the Kruskal–Wallis one-way analysis of variance. The association between pairs of variables was assessed by means of the Spearman rank-order correlation coefficient.

## RESULTS

### Pharmacokinetics and distribution of ^14^C-mTHPC

**Figure 1:**
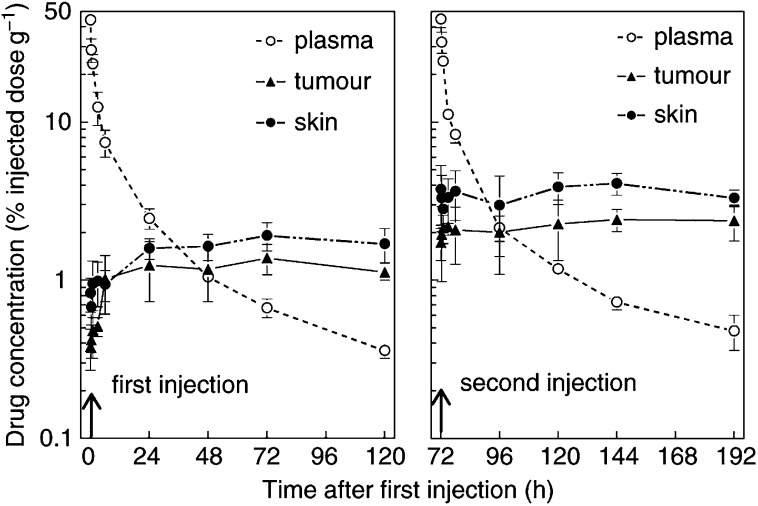
^14^C-mTHPC, as a percentage of the injected dose per gram tumour (▴) or skin (•) or per millilitre plasma (○), expressed as a function of time after a single drug injection (left panel) or two injections separated by 72 h (right panel). The results are means (±s.d.) of groups of six mice.

Pharmacokinetic profiles after single and double injections of ^14^C-mTHPC in tumour-bearing mice are shown in Figure 1

. The plasma levels decreased rapidly and the concentration–time curve was described by a second-order exponential decay, with *T*_1/2_ values of 1.3±0.4 and 20.9±3.1 h (mean±s.e.) after the first injection. *T*_1/2_ values for plasma clearance after the second injection were 1.1±0.3 and 17.8±1.7 h, which is not significantly different from those measured after a single drug dose. This agrees well with results of a separate study (unpublished results, not shown), in which the plasma concentration of nonlabelled Foscan was measured using HPLC. In those studies, the initial drug concentration in plasma of nude mice was 4079 ng ml^−1^, at 5 min after administration of a 0.3 mg kg^−1^ drug dose. This decreased to 82 ng ml^−1^ at 48 h, giving *T*_1/2_ values of 1.4±0.3 and 22.0±4.0 h.

Drug levels in both skin and tumour increased during the first 24 h after a single injection and then remained constant until 120 h. The data shown in Figure 1 are for samples taken from animals without saline perfusion, but separate experiments (not shown) demonstrated that perfusion to remove circulating blood did not significantly alter the measured drug levels in skin or tumour at any of the sampling times. After a second injection of ^14^C-mTHPC, there was a rapid increase in drug levels in both skin and tumour, with a steady state being maintained from 20 min to 120 h. These tissue drug levels were significantly higher than after the single drug dose (*P*<0.01 in all cases).

**Figure 2:**
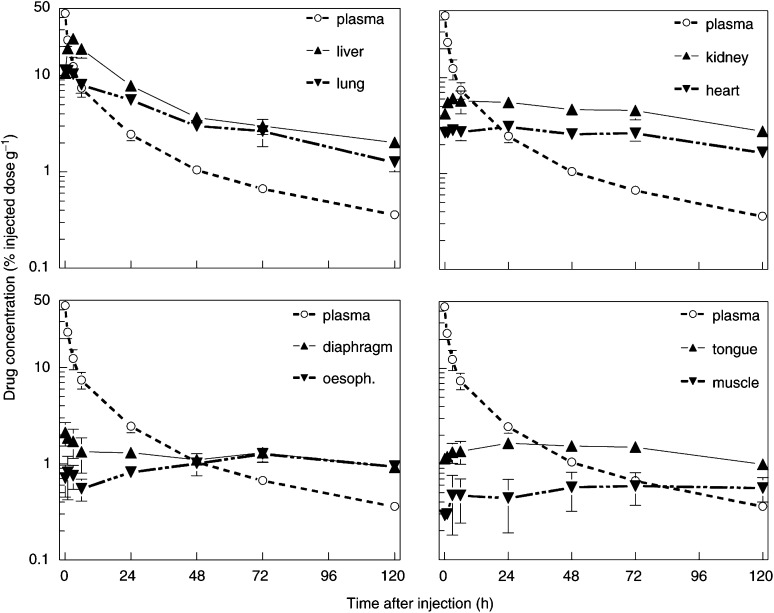
^14^C-mTHPC levels in other normal tissues (see text legends), as a function of time after a single drug injection. The plasma clearance curve, from Figure 1, is reproduced in each panel for comparison (○). Results are expressed as percentage of injected dose per gram tissue and are mean values (±s.d.) of six mice.

Drug levels in other tissues (from nonperfused mice) after a single drug dose are shown in Figure 2

. Peak drug concentrations in liver, lung, kidney and heart were all reached within the first 1–3 h, and these drug levels were much higher than was seen in the tumour at any time. Other organs had a more delayed uptake, similar to that seen in skin and tumour, and drug levels never exceeded 2% of the injected dose. Drug concentrations measured at 20 min after injection in perfused animals were lower than in nonperfused animals in lung, heart and kidney (data not shown). No other significant differences were seen between measurements made in perfused and nonperfused mice. There was a slow elimination of drug from liver and lung, with *T*_1/2_ values of 19.7±4 h and 18.2±43.5 h. This is comparable to the *T*_1/2_ values for plasma clearance, but lacks the initial fast clearance phase. Drug levels in all other tissues remained fairly constant, at near maximum levels, during the period 3–72 h.

### Tumour response to PDT

**Figure 3:**
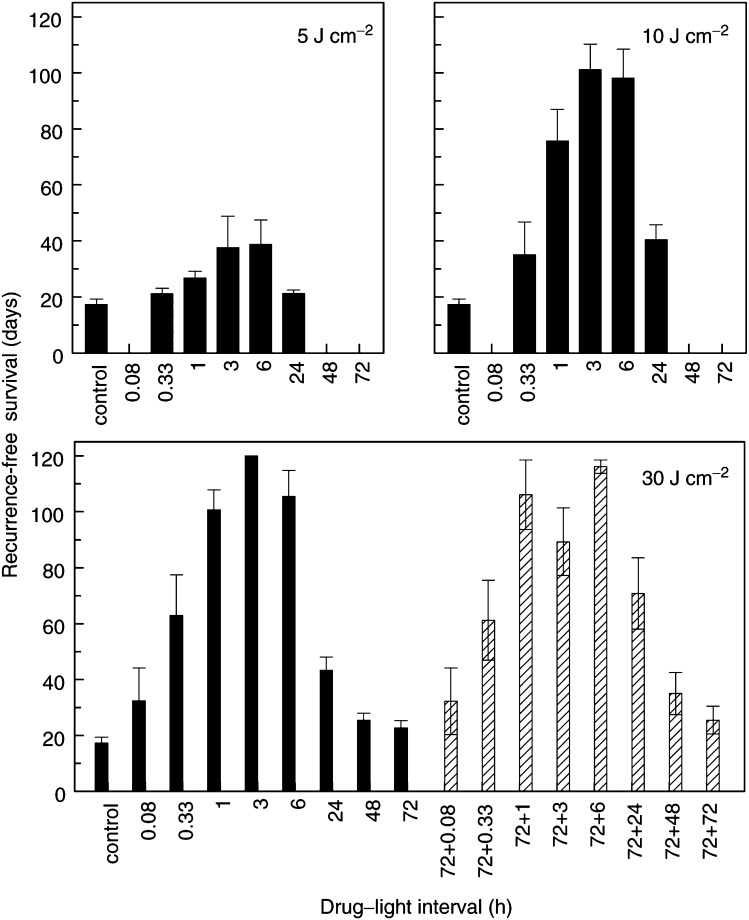
Mean recurrence-free survival (±s.d.) for groups of mice (*n*=8) illuminated with 5 or 10 J cm^−2^ at different intervals after a single Foscan dose (top panels), or with 30 J cm^−2^ after a single drug dose (solid bars, bottom panel) or two doses separated by 72 h (hatched bars, bottom panel). Cured tumours (see Table 2) were assigned a tumour-free survival of 120 days and were included in the calculation of mean-recurrence-free survival.

Tumour response (recurrence-free survival) was assessed for illumination with 30 J cm^−2^ at 5 min to 120 h after a single injection of Foscan (0.3 mg kg^−1^), or after two injections separated by 72 h (2×0.3 mg kg^−1^). These results demonstrated that illumination intervals of 1, 3 or 6 h were most effective (Figure 3

, bottom panel). There was no significant difference between these groups, either for illumination after a single drug dose or a double drug dose. At these times, a total of 70% (single injection) or 60% (double injection) of mice were cured of their tumours using a light dose of 30 J cm^−2^ (Table 2

**Table 2:** Number of cured^a^ tumours per treatment group for light doses of 5–30 J cm^−2^ applied at various intervals after one or two doses of Foscan

		**Drug–light interval**							
**Drug dose (mg kg^−1^)**	**Light dose (J cm^−2^)**	**5 min**	**20 min**	**1 h**	**3 h**	**6 h**	**24 h**	**48 h**	**72 h**
1×0.3	5	—	0/8	0/8	1/8	0/8	0/8	—	—
1×0.3	10	—	1/8	1/8	4/8	4/8	0/8	—	—
									
1×0.3	30	1/8	2/8	3/7^b^	8/8	5/8	0/8	0/8	0/8
2×0.3	30	1/8	1/8	5/7	3/8	5/7	1/7	0/8	0/8

a No palpable tumour at 120 days after treatment time.

b This group also included three tumours that did not regrow, but mice were sacrificed with lung metastases before the cure end point time of 120 days.

). Illumination at 1, 3 and 6 h after a single Foscan dose, or at 1 and 6 h after a double dose, was significantly more effective than illumination at all other intervals (*P*<0.05). Illumination at 3 h after the double dose was, however, not significantly different from 20 min (*P*=0.09) or 24 h (*P*=0.25). Illumination at intervals greater than 24 h was ineffective, with no cured tumours and very little growth delay compared with untreated tumours. There was no increase in tumour response for illumination after the double injection compared with the single injection and no significant differences between the two data sets, either within each drug–light interval or overall. Experiments with lower light doses applied at 20 min to 24 h after a single drug dose confirmed that the drug–light intervals of 3–6 h were the most effective (Figure 3, top panels and Table 2).

**Figure 4:**
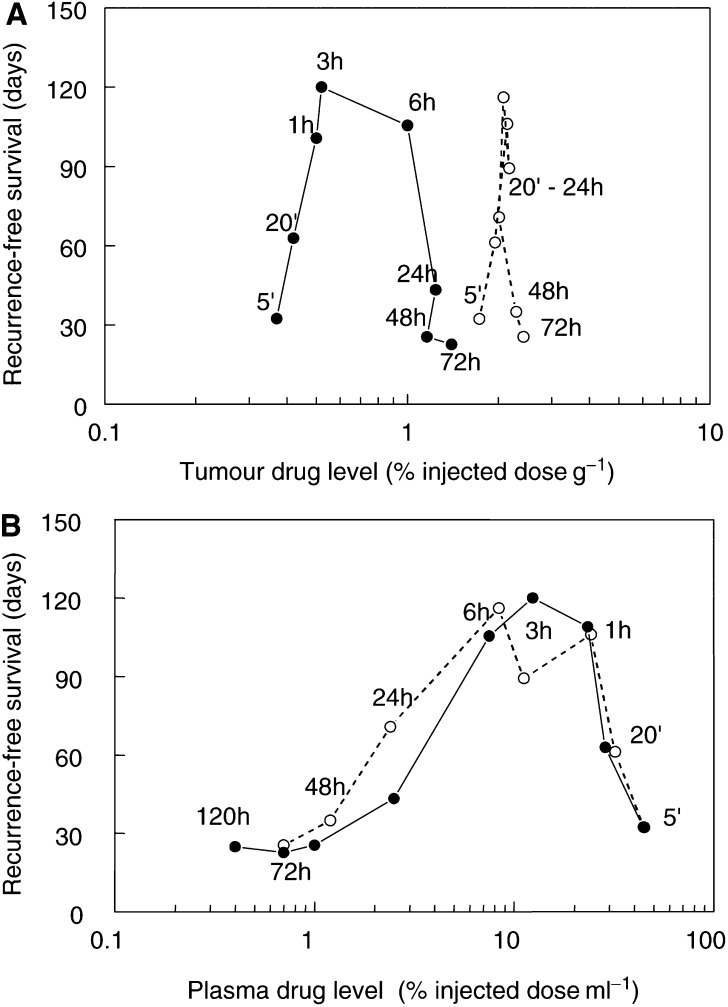
Mean recurrence-free survival for groups of mice illuminated at various times after a single Foscan dose (•) or two drug doses separated by 72 h (○). Results are expressed as a function of mean tumour drug levels (**A**), or mean plasma levels (**B**), measured in separate groups of mice, for each drug–light interval (written beside the data points).

Tumour response to PDT was evaluated as a function of drug levels in tumour and plasma at the time of illumination. After a single drug dose, the relation between response and tumour drug levels was described by a clockwise hysteresis (Figure 4A

), with a poor overall correlation between tumour drug levels and response (*P*=0.099). The PDT response increased with increasing drug concentration during the first 3 h, but for longer intervals the tumour response decreased markedly, whereas the drug concentrations continued to rise by almost a factor of 3 (0.5–1.4% of injected dose). For drug–light intervals >20 min, there was a *negative* correlation between tumour response and tumour drug levels (*P*=0.52). After a double injection of Foscan, there was a further increase in tumour drug levels, to a maximum of 2.0–2.4% of injected dose. The accumulation phase was much more rapid (20 min) and there was no correlation between response and tumour drug levels (*P*=0.779).

The relation between tumour response and plasma drug levels was described by a counterclockwise hysteresis (Figure 4B). During the initial distribution phase (20 min after a single or double drug injection), there was an inverse relation between plasma drug levels and PDT response. This indicates that the central blood compartment is not the effector compartment for Foscan-mediated PDT. At times beyond 1 h, the falling plasma concentrations were matched by decreasing tumour response to PDT. This pattern was repeated after the second injection. If all drug–light intervals (single and double injection) were considered, the correlation between tumour response and plasma drug levels was not significant (*P*=0.078). However, if the distribution times (20 min) were excluded, there was a highly significant correlation (*P*<0.0001) between plasma levels and tumour response.

### PDT skin response

**Figure 5:**
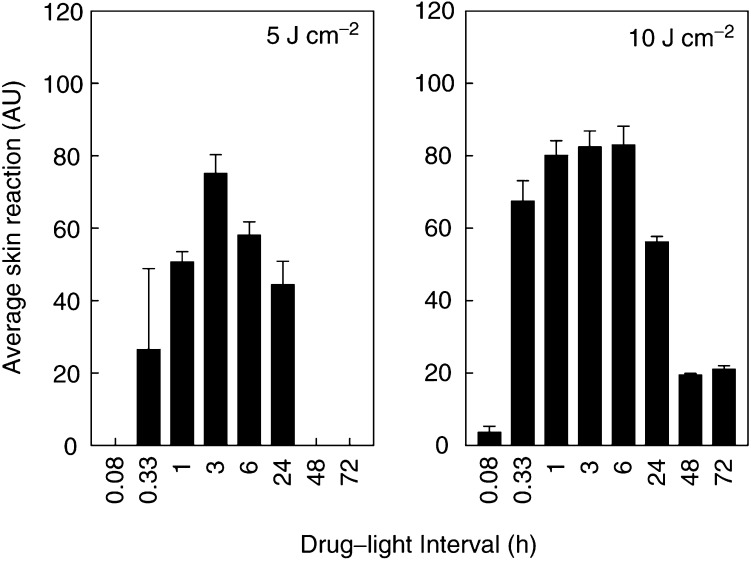
Mean skin reactions (±s.d.) for groups of mice (*n*=6) illuminated with 5 or 10 J cm^−2^ min to 72 h after a single Foscan dose.

**Figure 6:**
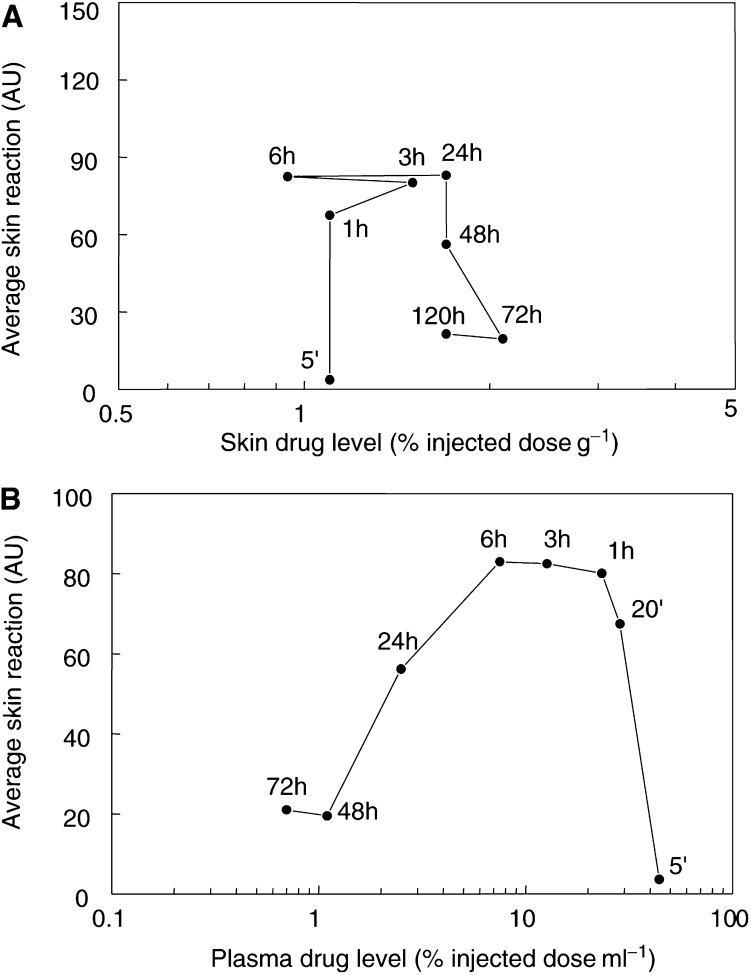
Mean skin reactions for illumination with 10 J cm^−2^ at different intervals after a single Foscan dose. Results are expressed as a function of mean skin drug levels (**A**), or mean plasma drug levels (**B**), measured in separate groups of mice, at each drug–light interval, as indicated.

PDT damage to normal skin was also assessed for illumination at 5 min to 72 h after a single dose of Foscan (0.3 mg kg^−1^). Skin reactions increased over the first 10 days after illumination, with healing thereafter. The average skin scores over the period 1–30 days were calculated and evaluated as a function of drug–light interval (Figure 5

). Neither illumination alone, nor illumination with an unsuitable wavelength of light at 24 h after Foscan, induced any measurable skin reaction. The PDT-induced skin damage was maximal for 10 J cm^−2^ at 1–6 h, with no significant difference between these three time intervals. Skin reactions for a drug–light interval of 20 min were slightly lower (*P* values ranged from 0.05 to 0.07) and reactions for all other intervals were significantly lower (*P*<0.004). There was no correlation between skin reactions and drug levels in the tissue at the time of illumination (*P*=0.570, Figure 6A

). The relation between skin reaction and plasma drug levels was described by a counterclockwise hysteresis (Figure 6B), similar to that seen for tumour response. The correlation between PDT response and plasma drug levels was, however, not significant (*P*=0.111 for illumination intervals 1–72 h).

## DISCUSSION

The primary objective of this study was to relate pharmacokinetic and pharmacodynamic parameters for the photosensitiser Foscan to the extent of PDT damage. Plasma drug clearance in these mice was biexponential, with *T*_1/2_ values of 1.3 and 20.9 h. This implies a rapid drug distribution into tissue compartments, as has been demonstrated in several other studies in both mice and rats (Morlet *et al*, 1995; Peng *et al*, 1995; Whelpton *et al*, 1996). The highest tissue drug levels were seen in liver and lung, followed by kidney and heart. These are all tissues containing reticuloendothelial cells, which are known to preferentially accumulate photosensitisers (Gomer and Dougherty, 1979). Elimination of drug from both tumour and normal skin was very slow, with near maximum levels being maintained until at least 120 h.

A comparison of the pharmacokinetic profiles with PDT responses in tumour and skin demonstrated major discrepancies between tissue drug loading and optimal illumination intervals for PDT efficacy. This was particularly apparent for the schedules with illumination after a double Foscan dose, where a plasma boost was given at times when tumour drug levels were already high. Tumour response in these mice varied from 85% cures to no significant growth delay, depending on the drug–light interval, whereas the tumour drug levels remained constant. There was a much closer correspondence between plasma drug levels and tumour or skin response, except for illumination during the first 20 min after drug delivery.

These results are in agreement with our earlier studies on Foscan-mediated response in a rapidly growing murine tumour RIF1 (Veenhuizen *et al*, 1997a). The previous study also demonstrated a very poor correlation between tumour drug levels and PDT efficacy, with a much better correlation for plasma drug levels. Once again, there was an initial distribution time of about 20 min, before illumination effected a PDT response. Other investigators have also reported a general lack of correlation between Foscan uptake in tissue and PDT effect, although most of these studies did not directly compare pharmacokinetics and PDT response (Ris *et al*, 1993; Morlet *et al*, 1995; Veenhuizen *et al*, 1997b).

One factor that could contribute to a decreasing PDT response, at longer illumination intervals, is tumour cell proliferation in the time between drug delivery and illumination. This could have a diluting effect on drug levels per cell and complicate interpretation of the results in rapidly growing tumours. Such an effect is, however, very unlikely to be a factor in the response of the slow growing H-MESO1 tumours, which have a volume doubling time of 15–17 days.

The lack of response in tumour and normal skin, illuminated during the first 20 min after Foscan (when plasma levels are maximum), indicates either that the circulating blood is not the effector compartment, or that the drug is initially in an inactive form (see below). If the endothelial cells in microvessels feeding the tumour represent the target population, as we propose, then a short delay before maximum tumour and tissue response to PDT would be reasonable. Several studies have used fluorescence microscopy to investigate Foscan distribution in tissues at increasing times after administration. Studies in mice transplanted with CaD_2_ tumours (Peng *et al*, 1995) showed strong fluorescence in the vascular walls of tumour vessels at 1 h after drug delivery, but low fluorescence in the tumour cells. At 24 h, strong fluorescence was seen in the cytoplasm of tumour cells, but not in the surrounding muscle cells. Results from studies using a Syrian hamster cheek pouch tumour model (Andrejevic-Blant *et al*, 1997) demonstrated high fluorescence levels in endothelial cells at 8 h after Foscan, with accumulation in squamous epithelial tissues from 2 to 4 days. Clinical studies in a group of patients treated with Foscan PDT for gynaecological cancers (Wierrani *et al*, 1997) or SCC of the upper airways (Andrejevic Blant *et al*, 2001) also demonstrated high fluorescence levels in endothelial and inflammatory cells during the first 20 h.

The results from this study demonstrated that Foscan PDT was relatively ineffective at controlling the regrowth of H-MESO1 xenografts when illumination was given at intervals of >24 h. This is in agreement with several other studies in rodent tumours and human xenografts grown in nude mice (Ris *et al*, 1993; Morlet *et al*, 1995; Van Geel *et al*, 1995; Veenhuizen *et al*, 1997a) , but it differs from clinical experience. Standard clinical protocols for Foscan PDT involve illumination with 20–30 J cm^−2^ at 4 days after a drug dose of 0.15 mg kg^−1^. Such schedules are clearly effective at controlling small superficial tumours at sites such as the oral cavity and upper aerodigestive tract (Dilkes *et al*, 1996; Grosjean and Savary, 1996; Fan *et al*, 1997; Savary *et al*, 1997) . However, very few clinical studies have systematically investigated the influence of changing the drug–light interval while keeping both drug and light dose constant. It is therefore not clear whether the 4-day illumination interval commonly employed represents the optimal treatment time. One clinical study in patients with multiple BCC of the skin did demonstrate significantly increased complete response (CR) rates for lesions illuminated at shorter intervals after a low dose of Foscan (0.1 mg kg^−1^) than for illumination at 3–4 days (Baas *et al*, 2001). The CR for lesions treated with 15 J cm^−2^ at 1–2 days was 83% (*n*=24), compared with a CR of only 37% for illumination at 3–4 days (*n*=27). These studies are now being extended to relate the response rate of BCC tumours to plasma and tumour drug levels at the time of illumination (Baas, unpublished data).

One possible explanation for the more persistent PDT efficacy seen for human tumours illuminated at >2 days after Foscan, may be the slower drug distribution and clearance. The initial rapid drug clearance phase, which is seen in rodents, does not occur in humans. Pharmacokinetic profiles for Foscan demonstrate an unusual delayed plasma peak at about 10 h after bolus injection, with subsequent elimination half-lives in the range of 30–60 h (Braichotte *et al*, 1995; Glanzmann *et al*, 1998). The plasma drug concentrations therefore remain at levels of at least 500 ng ml^−1^ for up to 96 h after a standard dose of 0.15 mg kg^−1^ Foscan. This contrasts with the pharmacokinetic profiles in rodents, which demonstrate peak plasma levels within a few minutes of drug administration and a rapid initial clearance, with *T*_1/2_ values of 1–3 h (Whelpton *et al*, 1995; Veenhuizen *et al*, 1997a). In the present study, plasma drug levels were only 1% of the injected dose at 48 h after administration of a 0.3 mg kg^−1^ Foscan dose. This is equivalent to a drug concentration of <100 ng ml^−1^, which was also confirmed in separate studies, where the Foscan plasma concentrations were measured by HPLC.

The reason for the delayed plasma peak in human plasma after Foscan injection is not fully understood. One contributing factor could be the protein-binding patterns of this drug, which differ from those of other photosensitisers. *In vitro* binding studies (Hopkinson *et al*, 1999) have shown that Foscan initially binds to a heavy, nonlipoprotein fraction (not albumin) in human plasma, which does not fluoresce, and subsequently redistributes to the plasma lipoproteins. Hopkinson and colleagues hypothesise that Foscan initially binds to this unknown protein fraction in a highly aggregated form and that it is subsequently taken up into tissue in this form. If true, this could explain why illumination during the initial period after Foscan injection is not effective. The rate at which the drug disaggregates and dissolves in the plasma lipoprotein fraction would then determine the minimum effective time for PDT.

In conclusion, these studies have demonstrated a pronounced disassociation between tumour drug levels of the photosensitiser Foscan and optimum drug–light intervals for PDT efficacy. We suggest that the tumoricidal effect of Foscan-mediated PDT is largely mediated via vascular damage and that the selectivity of the treatment is based on factors other than differential tumour drug uptake. This might also hold true for PDT using other photosensitisers, such as Photofrin, where vascular damage is an important component of the total effect. Plasma Foscan levels had a much greater influence on PDT response than either tumour or skin drug levels, although illumination during the first 20 min was relatively ineffective. If confirmed in clinical studies, this could offer opportunities for individual optimisation of treatment. Illumination could be given at intervals longer than that required for drug distribution, but before the plasma drug concentrations decrease below defined levels.
